# Integration of cytogenetic landmarks into the draft sequence of the human genome

**DOI:** 10.1038/35057192

**Published:** 2001

**Authors:** The BAC Resource Consortium, V. G. Cheung, N. Nowak, W. Jang, I. R. Kirsch, S. Zhao, X.-N. Chen, T. S. Furey, U.-J. Kim, W.-L. Kuo, M. Olivier, J. Conroy, A. Kasprzyk, H. Massa, R. Yonescu, S. Sait, C. Thoreen, A. Snijders, E. Lemyre, J. A. Bailey, A. Bruzel, W. D. Burrill, S. M. Clegg, S. Collins, P. Dhami, C. Friedman, C. S. Han, S. Herrick, J. Lee, A. H. Ligon, S. Lowry, M. Morley, S. Narasimhan, K. Osoegawa, Z. Peng, I. Plajzer-Frick, B. J. Quade, D. Scott, K. Sirotkin, A. A. Thorpe, J. W. Gray, J. Hudson, D. Pinkel, T. Ried, L. Rowen, G. L. Shen-Ong, R. L. Strausberg, E. Birney, D. F. Callen, J.-F. Cheng, D. R. Cox, N. A. Doggett, N. P. Carter, E. E. Eichler, D. Haussler, J. R. Korenberg, C. C. Morton, D. Albertson, G. Schuler, P. J. de Jong, B. J. Trask

**Affiliations:** 1 Department of Pediatrics, University of Pennsylvania, The Children's Hospital of Philadelphia, 3516 Civic Center Boulevard, ARC 516, Philadelphia, 19104 Pennsylvania USA; 2 Roswell Park Cancer Institute, Elm and Carleton Street, Buffalo, 14263 New York USA; 3 National Center for Biotechnology Information, National Library of Medicine, Building 38A/Room 8N805, Bethesda, 20894 Maryland USA; 4 National Cancer Institute, NIH, Building 10/Room 12N214, Bethesda, 20889-5105 Maryland USA; 5 The Institute for Genomic Research, 9712 Medical Center Drive, Rockville, 20850 Maryland USA; 6 Departments of Pediatrics and Human Genetics, Cedars-Sinai Medical Center, 8700 Beverly Boulevard, Los Angeles, 90048 California USA; 7 Computer Science Department, University of California Santa Cruz, 1156 High Street, Santa Cruz, 95064-1077 California USA; 8 Department of Biology, California Institute of Technology, Mail Code 147-75, Pasadena, 91125 California USA; 9 University of California San Francisco Cancer Center, Box 0808, San Francisco, 94143-0808 California USA; 10 Stanford University, Genome Lab, Mail Code 5120, Stanford, 94305-5120 California USA; 11 Sanger Center, Wellcome Trust Genome Campus, Hinxton, CB10 1SA Cambridge UK; 12 Fred Hutchinson Cancer Research Center, 1100 Fairview Avenue North C3-168, P.O. Box 19024, Seattle, 98109-1024 Washington USA; 13 Department of Molecular Biotechnology, University of Washington, Box 357730, Seattle, 98195-7730 Washington USA; 14 Departments of Obstetrics and Gynecology and Pathology, Brigham and Women's Hospital, Amory Lab Building 3rd floor, Boston, 02115 Massachusetts USA; 15 Department of Human Genetics, Case Western Reserve University, 10900 Euclid Avenue, Cleveland, 44106 Ohio USA; 16 Joint Genome Institute-Los Alamos National Laboratory, MS M888 B-N1, P.O. Box 1663, Los Alamos, 87545 New Mexico USA; 17 Joint Genome Institute-Lawrence Berkeley National Laboratory, 1 Cyclotron Road, Mail Stop 84-171, Berkeley, 94720 California USA; 18 Children's Hospital Oakland Research Institute, 747 52nd Street, Oakland, 94609 California USA; 19 Research Genetics, 2130 Memorial Parkway, Huntsville, 35801 Alabama USA; 20 Institute for Systems Biology, 4225 Roosevelt Way NE, Suite 200, Seattle, 98105-6099 Washington USA; 21 Department of Cytogenetics and Molecular Genetics, Women's and Children's Hospital, 72 King William Road, North Adelaide, 5006 South Australia Australia; 22 Computer Science Department, Howard Hughes Medical Institute, University of California Santa Cruz, 1156 High Street, Santa Cruz, 95064–1077 California USA; 24 Present Address: PanGenomics, 6401 Foothill Boulevard, Tujunga, California 91024 USA; 25 Present Address: Harvard Medical School, Cell Biology, 240 Longwood Avenue, Cambridge, Massachusetts 02115 USA; 26 Present Address: Gene Logic, Inc., 708 Quince Orchard Road, Gaithersburg, Maryland 20878 USA

## Abstract

We have placed 7,600 cytogenetically defined landmarks on the draft sequence of the human genome to help with the characterization of genes altered by gross chromosomal aberrations that cause human disease. The landmarks are large-insert clones mapped to chromosome bands by fluorescence *in situ* hybridization. Each clone contains a sequence tag that is positioned on the genomic sequence. This genome-wide set of sequence-anchored clones allows structural and functional analyses of the genome. This resource represents the first comprehensive integration of cytogenetic, radiation hybrid, linkage and sequence maps of the human genome; provides an independent validation of the sequence map^1,2^ and framework for contig order and orientation; surveys the genome for large-scale duplications, which are likely to require special attention during sequence assembly; and allows a stringent assessment of sequence differences between the dark and light bands of chromosomes. It also provides insight into large-scale chromatin structure and the evolution of chromosomes and gene families and will accelerate our understanding of the molecular bases of human disease and cancer.

**Supplementary information:**

The online version of this article (doi:10.1038/35057192) contains supplementary material, which is available to authorized users.

## Main

With the draft of the human genome available^2^, scientists can conduct global analyses of its gene content, structure, function and variation. One important challenge is to define the genetic contribution to human diseases. For many developmental disorders, inherited conditions and cancers, gross chromosomal aberrations provide clues to the locations of the causative molecular defects. These aberrations are visible as alterations in chromosomal banding patterns^3^ or in the number or relative positions of DNA sequences labelled by fluorescence *in situ* hybridization (FISH)^4^. Although tracing gross abnormalities to the level of DNA sequence^5^ has revealed the genetic causes of many diseases, molecular characterization of chromosomal aberrations has lagged far behind their discovery^6^. To proceed from cytogenetic observation to gene discovery and mechanistic explanation, scientists will need access to a resource of experimental reagents that effectively integrates the cytogenetic and sequence maps of the human genome.

**Figure 1:**
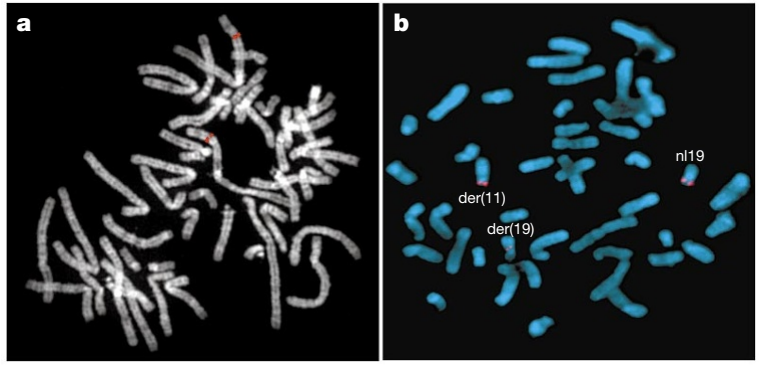
Cytogenetic analyses of sequence-integrated clones. **a**, Using FISH, fluorescent signals are observed at cytogenetic bands (grey) where fragments of a sequence-tagged BAC hybridize (red). **b**, Clones selected on the basis of band location were used in FISH analyses to map the breakpoint of a translocation involving chromosomes 11 and 19 in a patient with multiple congenital malformations and mental retardation (DGAP012, http://dgap.harvard.edu). Clone CTD-3193o13 spans the breakpoint on chromosome 19; red signal is split between the derivative chomosome 11 and derivative 19 chromosomes and is also present on the normal chromosome 19. The GTG-banded karyotype for this patient is 46,XY,t(11;19)(p11.2;p13.3).

We describe here the results of a concerted effort to assemble such a genome-wide resource of well mapped, large-insert DNA clones. Each clone has been localized directly to chromosomal band(s) by FISH (Fig. 1a) and assigned one or more unique sequence tags, which can anchor the clone to the emerging draft sequence. We used complementary strategies to amass the current set of 8,877 clones. The set, which consists primarily of bacterial artificial chromosome (BAC) clones, includes clones targeted to contain sequence-tagged sites (STSs) ordered along the genome by genetic linkage or radiation hybrid mapping (for well ordered and distributed coverage); clones randomly selected for end sequencing from the RPCI-11 library (for coverage of regions low in STSs); clones identified during intense mapping efforts that preceded sequencing of some chromosomes (for denser coverage); and clones suspected of being partially duplicated at more than one location in the genome (to flag regions of the genome that might complicate sequence assembly^7^). The molecular signatures are STSs (many corresponding to genes or expressed sequence tags (ESTs)), BAC end sequences, or the actual draft or final sequence of the clone (Table 1). Earlier publications have described genome-wide and chromosome-specific subsets of this collection^8,9,10,11,12^.

**Table 1:**
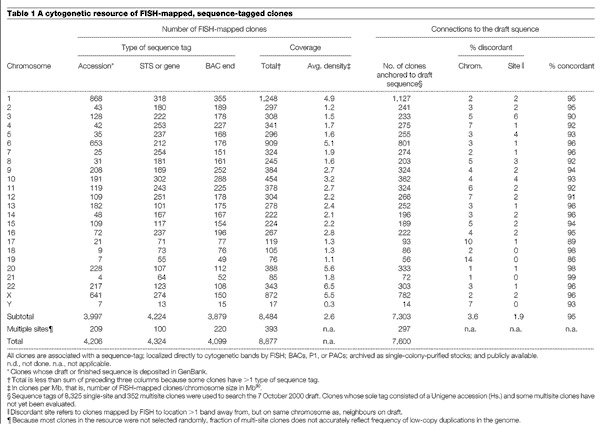
A cytogenetic resource of FISH-mapped, sequence-tagged clones

Each clone is publicly available as single-colony-purified bacterial stocks and is ready for distribution. Each clone can each be obtained from one of three stock centres by e-mail: mapped-clones@mail. cho.org, libraries@resgen.com and clonerequest@sanger.ac.uk. The website http://www.ncbi.nlm.nih.gov/genome/cyto provides information about all clones in this collection, including how to obtain each clone. (Additional information can be obtained at the websites listed in Supplementary Information 1).

The 8,877 clones provide excellent coverage of the human genome (Table 1), with at least one clone on average per megabase (Mb) for 23 of the 24 chromosomes. Clone density ranges from greater than ∼5 clones per Mb for chromosomes 1, 6, 20, 22 and X to about 0.3 clones per Mb for chromosome Y.

Our study provides an assessment of the representation of the human genome in the RPCI-11 BAC library^13^, which serves as the intermediate template for most sequencing efforts^2^ and the foundation of genome-wide contig assembly by fingerprint analyses^1^. We randomly selected 1,243 clones from this library for FISH analysis. The number of clones assigned to each chromosome correlated well with chromosome size, with no significant bias in the distribution of clones between Giemsa (G)-dark and G-light bands of chromosomes (see Supplementary Information 2 and 3).

Cytogenetic mapping is one of several methods that can produce a framework of ordered clones upon which the human sequence can be assembled. The resource provides an opportunity to cross-check these critical framework maps, because over 3,300 FISH-mapped clones have STSs that reference the radiation hybrid^14^ or linkage maps^15,16^. Overall, the concordance between cytogenetic map order and marker order established by radiation hybrid and linkage mapping is very high for clones with single cytogenetic locations (94–98%, depending on the map; Table 2). Significant discrepancies were observed for only around 140 of these clones and are probably due to errors in clone tracking. Integration of cytogenetic and linkage maps also aids efforts to map disease genes. The location of the cytogenetic abnormality in one patient can guide the choice of polymorphic markers to assess linkage in other families that have similar phenotypes, but no visible chromosomal aberrations.

**Table 2:**
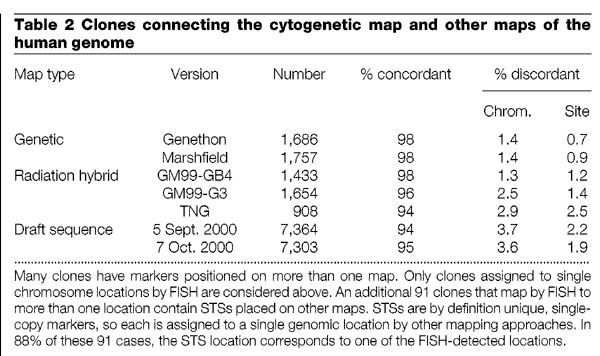
Clones connecting the cytogenetic map and other maps of the human genome

At present, 7,303 clones that map to single cytogenetic locations are positioned by their sequence tags on the draft sequence assembly of 7 October 2000 (Table 1). The fraction of clones located on the draft sequence ranges from 76% to 91% across different chromosomes (see Supplementary Information 4). We expect these percentages to rise as more sequence is merged into the draft and algorithms for locating tags are refined.

**Figure 2:**
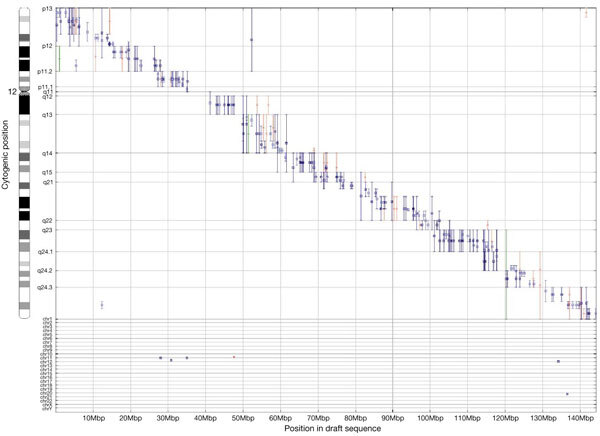
The correspondence between cytogenetic location and position on the 7 October 2000 draft sequence for chromosome 12. The band location of each clone is indicated by a range on the *y*-axis. Clones mapping to chromosomes other than 12 are indicated at the bottom. Colours differentiate assignments made in different laboratories. Each clone is anchored on the draft sequence by one or more sequence tags. Plots for the other chromosomes and the 5 September, 2000 assembly can be found at http://genome.ucsc.edu/goldenPath/mapPlots/. Genome browsers that assist researchers in navigating from cytogenetic location to other maps and detailed, annotated sequence information are available at http://www.ncbi.nlm.nih.gov/cgi-bin/Entrez/hum_srch (NCBI Mapviewer, which includes chromosomal aberrations associated with cancer and inherited disorders), http://www.ensembl.org/ and http://genome.ucsc.edu.

The connections between the cytogenetic map and the draft sequence are well distributed across the genome, and the correspondence in position on the two maps is excellent for these 7,303 clones (Fig. 2 shows chromosome 12 as an example). Of the 943 contigs of overlapping clones in the 7 October 2000 draft sequence, 660 are connected to the cytogenetic map by at least one clone, and 531 by two or more clones. Thus, many contigs can be oriented on the chromosome on the basis of FISH results of constituent clones. Relatively few discrepancies between cytogenetic location and position in the draft sequence are apparent at this level of resolution (∼5% of the clones map either to other chromosomes or more than one band away from the expected position; Table 1). We found only eight locations where the cytogenetic data indicated that portions of the sequence were misplaced within an earlier draft assembly (5 September 2000). The sequencing centres used these cytogenetic findings to locate errors in the assembly and produce the later draft of improved quality (Table 2).

FISH analyses of this clone collection reveal abundant paralogous relationships among sites dispersed across the human genome. Of 1,243 clones randomly selected from the RPCI-11 library, 5.4% hybridize to more than one chromosomal location (see Supplementary Information 3). The entire collection includes 393 clones that together identify over 150 bands containing at least one segment with significant homology to one or more (up to 25) other sites in the genome (see Supplementary Information 5). These data provide clues to duplications and exchanges that have occurred within and between chromosomes. Among the 393 clones, 111 contain blocks duplicated within the same chromosome; 282 hybridize to more than one chromosome. Paralogous relationships involving pericentromeric and subtelomeric regions of multiple chromosomes are particularly frequent and complex. Clones in the collection also identify low-copy duplications specific to chromosomes 1, 7, 11 and 16, the pseudoautosomal regions of X and Y, and sites of the olfactory receptor gene family^17^. Many previously undescribed patterns were also observed; some were confirmed with two or more clones, but others require further study to verify that they reflect true duplications.

Many of these duplications are functionally significant, as some have generated multigene families, and some are potential sites of recombination events, which can result in chromosome abnormalities. The cytogenetic data should greatly facilitate analyses of these regions, which are likely to pose challenges to sequence assembly. The sequence tags of 84% of the clones that hybridize to more than one site were placed in the 7 October 2000 draft assembly, and the location(s) were roughly consistent with at least one FISH observation for 88% of these clones. Collectively, the multisite clones highlight regions that are more likely to become entangled with other regions of the genome during sequence assembly than clones with single FISH locations. Indeed, global BLAST analyses show that regions encompassing sequence tags of multi-site clones (either the sequence of the FISH-mapped clone or a surrogate clone from the assembly) contain blocks of homology found at an average of around 3.9 chromosomal locations (compared to around 1.3 for the regions underlying clones with single FISH signals). The regions observed by FISH and revealed through homology searches are not fully congruent, however (not shown). These findings indicate that both FISH and sequence analyses may underestimate large-scale duplications and that these complex, inter-related regions of the genome will require special attention during the finishing stages of genome sequencing.

The extensive integration of cytogenetic and primary sequence data gives investigators access to fine-structure information—including details on predicted genes—for cytogenetic locations of interest. Tools such as NCBI's MapViewer and the UCSC and ENSEMBL genome browsers (see Fig. 2 for URLs) allow researchers to navigate readily between chromosomal location and annotated sequence.

This integration provides insight into the sequence differences underlying cytogenetic banding patterns. Sequence analyses of 200-kilobase (kb) regions surrounding the sequence tags of 338 clones mapped with the finest band resolution reveal more striking differences in the base-pair composition between Giemsa-positive and -negative bands than were predicted from earlier studies^18^. These clones were mapped with high precision to 850-level bands of varying staining intensity^19^ on seven chromosomes. The AT content of 58 of the 59 clones in the darkest G-bands exceeds the genome-wide average of 0.59 (mean 0.63), whereas the AT content of only 22 of the 143 clones in G-negative bands is higher than average (mean 0.55; χ^2^ = 43, *P *< 0.005). These data confirm that dark G-bands are more AT-rich than G-negative bands.

The utility of a sequence-integrated cytogenetic resource is illustrated by two examples. In the first, clones are applied in conventional FISH assays to rapidly narrow the search for candidate genes disrupted or deregulated by translocations causing developmental disorders. The process is expedited by selection of clones assigned to the regions implicated by banding analyses. In a patient with multiple congenital malformations and mental retardation (DGAP012, http://dgap.harvard.edu), a breakpoint-spanning clone was identified (Fig. 1b). This clone spans a 170-kb interval containing the gene for MKK7, a human mitogen-activated protein kinase, and a novel sequence with homology to the tre-2 oncogene, both plausible candidate genes. More typically, breakpoints will be mapped to an interval between neighbouring clones. For example, a translocation implicated in mental retardation in another patient maps to an interval containing at least 12 genes, including protocadherin 8, a promising candidate given its exclusive expression in fetal and adult brain^20^.

**Figure 3:**
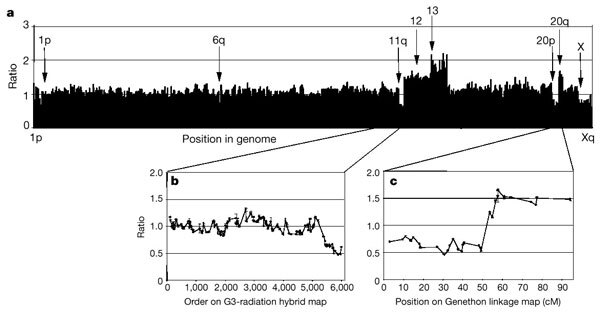
Copy-number analysis of myeloblastic leukaemia ML-2 cell line using CGH and a genome-wide array of around 2,000 BAC clones. The ML-2 cell line has acquired chromosomal abnormalities in addition to those present in the original tumour during long-term culture. CGH maps regions of abnormal copy number by comparing the relative efficiency with which test (Cy3-labelled ML-2 DNA) and reference (Cy5-labelled normal female DNA) hybridize to clones on the array. The array excludes clones that hybridize to multiple sites in the genome. **a**, Fluorescence ratios of Cy3 to Cy5 fluorescence for each BAC normalized to the median ratio for all 2,000 clones on the array, ordered from 1pter to Xqter. Arrows, chromosomal regions showing significant copy number variations. The lower ratio on the X indicates expected ratio for mismatched sex of test and reference DNAs. Fluorescence ratios of clones on chromosomes 11 (**b**) and 20 (**c**) are shown with clones ordered according to position of their STSs on the G3 radiation hybrid or Genethon linkage maps, respectively.

In the second example, an array of around 2,000 BAC clones from the collection is used to perform a genome-wide scan for segmental aneuploidy by comparative genomic hybridization (CGH) (Fig. 3 and A. Snijders *et al.*, in preparation). The array format offers better sensitivity and resolution^21,22^ than metaphase chromosomes, the traditional target for CGH^23^, and, because the arrayed clones are integrated into the draft, copy-number abnormalities can be related directly to sequence information. To illustrate the power of array CGH, the ML-2 cancer cell line was ‘karyotyped’ using the array. Array CGH revealed relative copy-number losses on 1p, 6q, 11q and 20p and gains of 12, 13 and 20q (Fig. 3). Copy-number abnormalities on chromosomes 6, 11 and 20 were subsequently confirmed by FISH using clones predicted by array CGH to be included in the region of loss. Several of these alterations were noted in previous banding analyses (1p-, 6q-, 11q-, +12, +13q+)^24^, but array CGH locates the breakpoints precisely relative to BACs that reference specific locations in the sequence.

More than 7,500 clones now link the cytogenetic map and sequence of the human genome. Application of these reagents in combination with increasingly detailed knowledge of genes and other functional motifs in the human sequence will transform the process of identifying genes that are altered in cancer and other diseases. Ultimately, this resource will contribute to a better understanding of the organization of the cell nucleus, the compacting of DNA into mitotic chromosomes, and the basis of the chromosomal banding patterns that have been so valuable in uncovering the aetiology of human diseases.

## Methods

GenBank was screened for draft, finished or end sequences derived from clones in this collection. BACs were screened for STS content by a combination of hybridization and polymerase chain reaction (see refs 8, 25 and Supplementary Information for details). Sequence tags were located on the draft sequence by a combination of methods (see Supplementary Information and refs 26, 27). Sequence at these locations was compiled with the results of a genome-wide BLAST analysis (ref. 2 and J. A. Bailey and E. E. Eichler, in preparation) to identify paralogous regions of the genome (regions in the draft sequence containing ∼ 20 kb of sequence that match sequence of the FISH-mapped clone or that of a surrogate clone from the assembly at ∼ 90% identity in non-repeat-masked bases over each 1-kb segment), and these locations were translated into estimated band positions using a dynamic programming algorithm (T. S. Furey *et al.*, in preparation; and see Supplementary Information).

Details of FISH procedures are provided elsewhere^4,28^. Only locations of unique or low-copy portions of the clone are identified, because high-copy interspersed repetitive sequences were suppressed by addition of unlabelled *Cot1* DNA. Replicate analyses indicate that the precision of FISH assignments to metaphase bands is roughly 5–10 Mb (1–1.5 band). A subset of 442 clones was ordered at very high (∼2–3-Mb) resolution^11^. FISH analyses were performed using DNA from the bacterial stock used for STS typing. Data that failed to replicate (for example, replicate FISH analyses of the same clone or different clones assigned the same marker) have been removed. Hybridization to arrays was carried out as described previously^29^ and by Snijders *et al.* (in preparation).

## Supplementary information

Supplementary Information comprising; Methods, Links, 2 Figures and a Table.
