# Supplementary material for: Integration of cytogenetic landmarks into the draft sequence of the human genome
Source: Nature. 2001;409(6822):953–8. doi: 10.1038/35057192 (PMC7845515; doi:10.1038/35057192)
Supplement: Supplementary file 1 — Supplementary Information comprising; Methods, Links, 2 Figures and a Table. [file 41586_2001_BF35057192_MOESM1_ESM.doc]

**Supplementary Information for Trask et al.**

## METHODS

Details of FISH procedures are provided elsewhere^1^. Typically, clones were biotinylated by nick translation, hybridized to cytogenetic preparations of metaphase cells, and detected with fluorescein-conjugated avidin. Only locations of unique or low-copy portions of the clone are identified, because high-copy interspersed repetitive sequences in the probe were suppressed by the addition of unlabelled Cot1 DNA. Banding patterns were produced using DAPI or chromomycin/distamycin^2^. Replicate analyses indicate that the precision of FISH assignments to metaphase bands is roughly 5-10 Mbp (1-1.5 band). The sensitivity of FISH in these assays is estimated to be around 20 kb. A subset of 442 clones was ordered at very high (~2- to 3-Mbp) resolution^3^. Among the collection is a specially curated set of 1243 clones for which FISH and end-sequencing were performed on the same samples. End-sequences were repeated and confirmed for 806 of these clones^4^. FISH was repeated using a separate single-colony isolate for each clone in this curated set that was observed to hybridize to more than one genomic location.

Hybridization to arrays was carried out as described previously^5,6^ and by A. Snijders et al (in preparation).

BACs were screened for STS content by a combination of hybridization and PCR. For example, at the University of Pennsylvania, mapping of RPCI-11 clones to STS markers followed a four-step procedure consisting of two rounds of filter hybridization (the first employing a multiplexed set of ~15 probes) and two rounds of PCR verification (the second using glycerol stocks of single colony isolates as template)^7,8^. A high-throughput screening strategy based on “Overgo” hybridization probes was used at Roswell Park Cancer Institute. Pairs of 24-mer oligonucleotides sharing 8 bp of complementary sequence at their 3’ ends were designed from the sequences of STS markers, annealed, and their overhangs filled in with 32^P^-dCTP and 32^P^-dATP using Klenow. Complex probes were generated using a 6x6x6 three-dimensional pooling scheme and hybridized to high-density RPCI-11 BAC filters providing 6-fold genome coverage. Hybridization results were digitized, analyzed, and scored with ArrayVision (Imaging Research, St. Catherines, Canada), and deconvoluted with the IDProbes program (Gregory Caldwell, RPCI). Hybridization-positive clones were confirmed by PCR using commercially available and custom-designed primer sets.

Sequence tags were located on the draft sequence assemblies of September 5, 2000 and October 7, 2000 (final assembly from January 9, 2001), by a combination of methods. Primer sequences of STS were located by using “electronic PCR” with stringent parameters that allowed a maximum of a 10-bp difference in the size of expected product and one mismatch in each primer sequence, but not within 7 bp of the 3’end^9^. STS hitting more than 6 sites in the draft were not used. BAC-end sequences of cytogenetically mapped clones were obtained from the dbGSS division of GenBank, masked of low-complexity regions and interspersed repeats with RepeatMasker, and aligned to the draft sequence Megablast^10^. Alignments of >100bp and >95% identity were retained. Ends that aligned to >6 genomic sites were discarded. For ends aligning to 2-6 sites, the longest and best match was selected. Clones with sequenced inserts were localized on fingerprint contigs using their GenBank accession numbers. Clones were flagged if they spanned more than 1 Mbp. The results for unambiguously placed clones were used to generate the values reported in Table 1 for the clones with single FISH sites, Figure 2 (and the plots referenced in Fig. 2 legend), and Supplemental Information 4. Ambiguity in placement was allowed for clones hybridizing by FISH to >1 site. To evaluate the paralogous relationships among the regions identified by the clones, sequenced inserts of clones were also placed on the preliminary 10 November 2000 assembly of the 7 October 2000 draft sequence using Blast after masking for repeats. Blast hits of 95% identity and >10 kb in length were selected for placing clones on the genome; if two different blast hits were less than 265 kb apart on a contig, they were merged to join hits that may have been separated by repeatmasking. 7452 single-site and 298 multi-site sequence-tagged clones were placed on the October 7 draft sequence in 8431 and 410 locations, respectively, by these combined methods. If the sequence-tagged clone was one of the sequenced clones used in the construction of the draft, then the placement consisted of this clone itself, otherwise a surrogate clone was chosen from among those clones used to construct the draft that overlap with the sequence-tagged site(s) identified for the clone to define the placement. We then compared these placements to a list of regions identified to contain paralogous segments as a result of a genome-wide BLAST analysis (Ref. 12; and J. A. Bailey and E. E. Eichler, in preparation) using a threshold set to detect regions >1 kb with >90% identity. Paralogous regions corresponding to each placement were identified as regions in the draft sequence that have at least 20 kb of sequence that matches sequence in the placement with at least 90% identity in non-repeat-masked bases over each 1-kb part. Paralogous regions were merged if within 200 kb of each other. Counting the original placements, this analysis resulted in 9435 and 1154 paralogous regions for the single-site and multi-site sequence-tagged clones, respectively. In order to compare these locations to FISH observations, they were translated into estimated band positions using a dynamic programming algorithm, the position of all clones mapped onto the assembly and localized by FISH to single bands, and band lengths from ISCN. The dynamic programming method minimizes the number of clones that are discordant in their band predictions, subject to the constraint that the bands must be in the proper order (T.S. Furey et al., in preparation).

Supplemental Notes:

An additional 3076 FISH-localized, sequence-tagged BACs exist for chromosome 5 (http:/www.jgi.doe.gov/) but these clones are not tallied for this report, because their band assignments were indirectly inferred from fractional length measurements. Cosmids mapped to chromosome 19 (http://www-bio.llnl.gov/bbrp/genome/html/

chrom_map.html) are not included here due to their relatively small insert size. Roughly 500 FISH-mapped clones are also excluded from consideration, because they lacked sequence tags. The FISH-analyzed clones are only a small fraction of the clones assigned STSs in the course of this study (http://genomics.med.upenn.edu/

genmapdb/ and http://genomics.roswellpark.org/human/overview.html.

# References

1. Trask, B.J. in: Genome Analysis: A Laboratory Manual. Cold Spring Harbor Laboratory Press, Cold Spring Harbor NY . 4: 303-413 (1999).
2. Korenberg, J.R., Chen, X.-N. Human cDNA mapping using a high-resolution R-banding technique and fluorescence in situ hybridization. Cytogenet. Cell Genet. 69: 196-200 (1995).
3. Kirsch, I.R. et al. A systematic, high-resolution linkage of the cytogenetic and physical maps of the human genome. Nature Genet. 24: 339-340 (2000).
4. Zhao, S. et al. Human BAC ends quality assessment and sequence analyses. Genomics 63: 321-332 (2000).
5. Pinkel D. et al. High resolution analysis of DNA copy number variation using comparative genomic hybridization to microarrays. Nature Genet. 20: 201-211 (1998).
6. Albertson, D.G. et al*.* Quantitative mapping of amplicon structure by array CGH identifies CYP24 as a candidate oncogene. *Nature Genet.* 25: 144-146 (2000).
7. Cheung, V.G. et al. A resource of mapped human bacterial artificial chromosome clones. Genome Res. 9: 989-993 (1999).
8. Morley, M. et al. GenMapDB: A database of mapped human BAC clones. *Nucleic Acids Res.* in press (2000).
9. Schuler, G.D. Electronic PCR: bridging the gap between genome mapping and genome sequencing. Trends Biotechnol. 16: 456-459 (1998).
10. Zhang, Z., Schwartz, S., Wagner, L., Miller, W. A greedy algorithm for aligning DNA sequences. J. Comput. Biol. 7: 203-214 (2000).
11. The International Human Genome Sequencing Consortium. Initial sequencing and analysis of the human genome. Nature (in press).

Links to information on resources for cytogenetic analyses of the human genome

# GENOME-WIDE RESOURCES

Resource overview (NCBI) – <http://www.ncbi.nlm.gov/genome/cyto>

BAC resource (UW/FHCRC) - <http://fishfarm.fhcrc.org/bacresource/index.shtml>

Cancer Chromosome Aberration Project (CCAP) - <http://www.ncbi.nlm.nih.gov/CCAP/>

CSMC Collection - <http://www.csmc.edu/genetics/korenberg/projects.html>

ENSEMBL genome browser – <http://www.ensembl.org/>

GenMapDB (U.Penn.) - <http://genomics.med.upenn.edu/genmapdb/>

Molecular Genetic Resources (U.Bari) - <http://www.biologia.uniba.it/rmc/>

NCBI MapViewer - <http://www.ncbi.nlm.nih.gov/cgi-bin/Entrez/hum_srch>

Oncogene BACs (Caltech) - <http://informa.bio.caltech.edu/Bac_onc.html>

Roswell Park Cancer Institute - <http://bacpac.med.buffalo.edu/human/overview.html>

Sanger Center - <http://www.sanger.ac.uk/HGP/Cytogenetics/>

UCSC genome browser – [http://genome.ucsc.edu](http://genome.ucsc.edu/)

# EXAMPLES OF CHROMOSOME-SPECIFIC RESOURCES

Chromosome-5 BACs (LBNL) - <http://www-hgc.lbl.gov/human-maps.html>

Chromosome-16 BACs (LANL) -
<http://jgi-lanlpublic.lanl.gov:80/Chr_16_mapping_Home/chr16map.html>

Chromosome-19 clones (LLNL) - <http://www-bio.llnl.gov/bbrp/genome/html/chrom_map.html>

# WHERE TO OBTAIN CLONES

BACPAC resources (CHORI) - http://www.chori.org/bacpac/; email: [mapped-clones@mail.cho.org](mailto:mapped-clones@mail.cho.org)

Research Genetics - http://www.resgen.com/resources/index.php3; email: [libraries@resgen.org](mailto:libraries@resgen.org)

Sanger Center - <http://www.sanger.ac.uk/HGP/Cytogenetics/> ; email: [clonerequest@sanger.ac.uk](mailto:clonerequest@sanger.ac.uk)

Distribution of 1176 randomly selected RPCI-11 BACs that hybridize by FISH to single locations in the human genome. The chromosomal distribution suggests that the library samples the euchromatic portion of the genome relatively uniformly. The expected number of clones per chromosome is the total number of clones (1176) divided by estimated chromosome size (from Trask et al., 1989). The values for X and Y are halved to correct for the fact that the library was produced from DNA from a male and sequences from the X and Y are expected to be present at half the frequency of autosomal sequences.


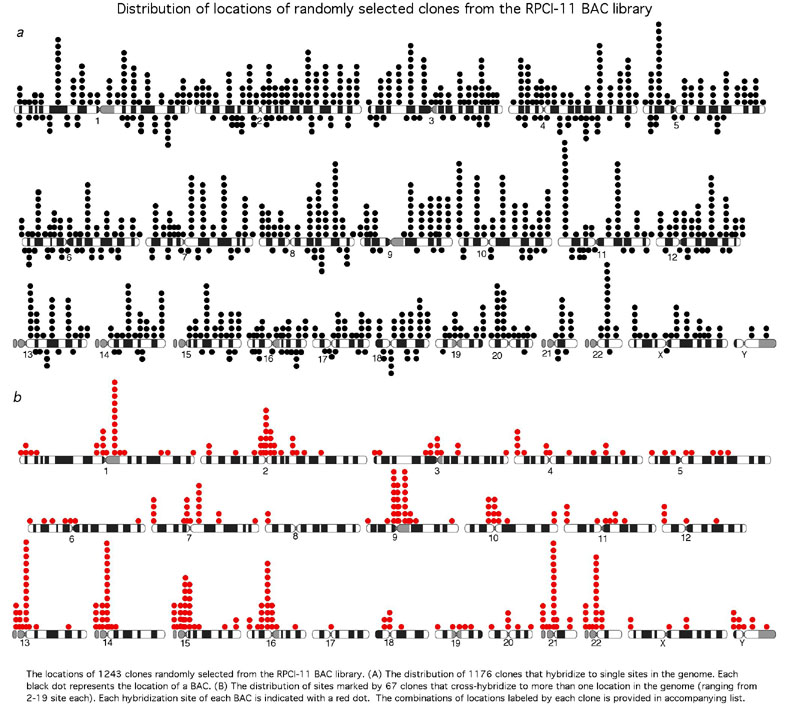


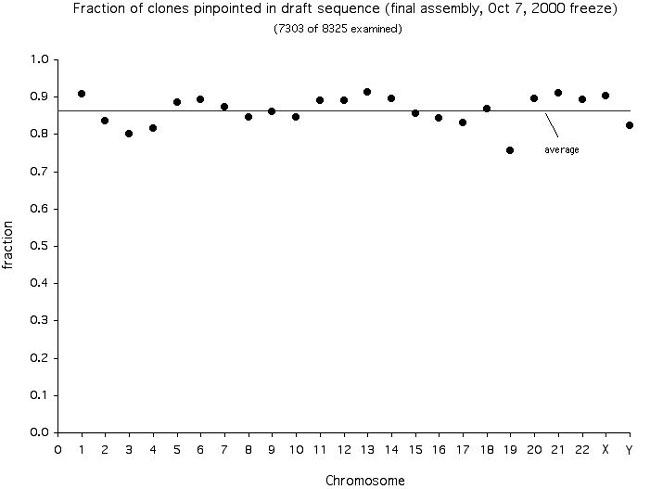


| **393 large-insert clones in the cytogenetic resource that hybridize by FISH to > 1 sites in in the genome** | | |  |  |  |
| --- | --- | --- | --- | --- | --- |
| Clone | RandomSet? | FISH sites (lab) | |
| CTA-246H3 |  | 11q13.1-11q13.5,22q11.21-22q12.2(SC) | |
| CTA-338m12 |  | 5q35,19p13.3(UWMBT) | |
| CTA-923A4 |  | 16q23,16p12-16p13.1,16p12.3-16p13.1(LANL) | |
| CTB-13D16 |  | Yp11.3,Xp22.1(CSMC) | |
| CTB-13M4 |  | 3p21.3,3p23(CSMC) | |
| CTB-18p10 |  | 7q22,7q32(CSMC) | |
| CTB-22F17 |  | 1p31.2,1p22(CSMC) | |
| CTB-53P14 |  | 11q23.3-11q24,11q23.1(CSMC) | |
| CTB-7H23 |  | 7q22,7q11.23(CSMC) | |
| CTC-216H12 |  | Xp22,22q12(SC) | |
| CTC-800g3 |  | 16q24,19p13.3,15q26,1p36,1q44,7p22,Yq11.2,6p25,5q35,3q29,6q27,7q36,9q34,20p13,12p13,11p15(UWMBT) | |
| CTC-802f4 |  | 4q26,16q24,19p13.3,6q27,20p13,20q13.3,1q42,7p11.1,11p15,1p36,1q44,7p22(UWMBT) | |
| CTD-2004F18 |  | 18p11.3,9q34(UWMBT) | |
| CTD-2005N23 |  | 7p11.1-7p11.2,7q11.1-7q11.21(UWMBT) | |
| CTD-2011F16 |  | 1p12-1p13,1q12-1q21(UWMBT) | |
| CTD-2065P18 |  | 19p13.2,19q13.2(UWMBT) | |
| CTD-2184G2 |  | 8p23,6p25,5q35,19p13.3,15q26,3q29,11p15,6q27,1p36(UWMBT) | |
| CTD-2189I5 |  | 16q24,6q27,13q14,1p36,12q24.3,7p22(UWMBT) | |
| CTD-2220h21 |  | 16p13.3,16q24,16p11.1-16q11.1,19p13.3,1q41,1q43,1p36,7p22,Yq11.2,2q37,4q26,6p25,8p23,5q35,3q29,6q27,9q34,10p11.1-10q11.1,11p11.1-11q11,20p13,20q13.3(UWMBT) | |
| CTD-2237k2 |  | 16q24,19p13.3,1q42,7p12,1p36,1q44,7p22,Yq11.2,2q37,4q26,7q32,5q35,3q29,8p23,9q34,10p11.1-10q11.1,20p13,20q13.3,11p15(UWMBT) | |
| CTD-2243O22 |  | 16p13.3,13q34,2q13-2q14,16q24,19p13.3,15q26,17q25,1q42,1p36,1q44,7p22,Yq11.2,5p15.3,2q37,4q26,6p25,7q32,8p23,5q35,3q29,7q34,6q27,9q34,10p11.1-10q11.1,20p13,11p15,11q25,14q32(UWMBT) | |
| CTD-2371D6 |  | 16q24,19p13.3,18q23,15q26,7p11.1,1q42,7q11.1,1p36,1q44,7p22,4q26,6p25,8p23,5q35,3q29,6q27,9q34,10p11.1-10q11.1,20p13,10p15,11p15(UWMBT) | |
| CTD-2503F15 |  | Yq11.2,6p25,4q26,5q35,19p13.3,3q29,6q27,9q34,20p13,1q41,14q21,1p36,7p22(UWMBT) | |
| CTD-2589I9 |  | 8p23,9q34,1p36(UWMBT) | |
| CTD-2607H18 |  | 2q13-2q14,16q24,19p13.3,15q26,7p11.1,1q42,7q11.1,2p25,1p36,1q44,7p22,4q26,6p25,7q32,8p23,5q35,3q29,6q27,9q34,10p11.1-10q11.1,20p13,12p13,20q13.3,11p15(UWMBT) | |
| CTD-3006C19 |  | 5p15.3,2q13-2q14,6p25,4q26,5q35,9p24,9q34,11p15,7p11.1,7q11.1,1p36,1q44,7p22(UWMBT) | |
| CTD-3027O20 |  | 17q21,16p13.3,16q24,19p13.3,1q42,1p36,7p22,2q37,6p25,3q29,6q27,10p11.1-10q11.1,20p13,12p13,20q13.3,11p15,10q26(UWMBT) | |
| CTD-3043C11 |  | 8p23,2q37,3q29,1p36(UWMBT) | |
| CTD-3069P12 |  | 8p23,6p25,5q35,15q26,10q26(UWMBT) | |
| CTD-3186O7 |  | 15p11.1-15q11.1,8p23,6p25,15q25,5q35,3q29,15q26,6q27,21p11.1-21q11.1,22p11.1-22q11.1,11p15,1p36,6p22(UWMBT) | |
| RP1-101A2 |  | 20q12-20q13.13,6q22(SC) | |
| RP11-101E9 |  | 7q22,7q11.23(UWMBT) | |
| RP11-102O5 |  | Yq11.2,2q37,16q24,5q35,15q25,19p13.3,6q27,1q41,4q11,1q43,1p36(UWMBT) | |
| RP11-104F9 |  | 2p11.1-2q11.1,20p11.1-20p11.2,3p11,2q12,22q11.2,7p11.1-7p11.2(UWMBT) | |
| RP11-105L10 |  | Yq11.23,Yp11.2(UWMBT) | |
| RP11-106K13 |  | 4q35,21q11.2(UWMBT) | |
| RP11-107C1 |  | 8q24.2,8q21.1(UWMBT) | |
| RP11-107P20 |  | 16p11.1-16p12,16p13.2,18p11.2(UWMBT) | |
| RP11-109H7 |  | 18q22.1,14q11.2(UWMBT) | |
| RP1-110C15 |  | 2p11.1-2p11.2,Xq27.1-Xq27.3(SC) | |
| RP11-111A8 |  | 17p11.2,13q33(UWMBT) | |
| RP11-112L19 |  | Yp11.3,Xp22.3(UWMBT) | |
| RP11-114G11 |  | 7p11.2,7q11.2(UWMBT) | |
| RP11-115E20 |  | Yp11.3,Xq21(UWMBT) | |
| RP11-115M14 |  | 1q24.1-1q25.2,1p31.2-1p32.1(SC) | |
| RP11-115N23 |  | 5q11.1-5q11.2,1p11.1-1p13.2(SC) | |
| RP11-115O15 |  | 9p12-9p21.1,9q13,9q22.2-9q31.1(SC) | |
| RP11-118J22 |  | 10q21.1,2q13(SC) | |
| RP11-119M6 |  | 4q35,21q11.2,13p11.1-13p11.2,14p11.1-14q11.1(UWMBT) | |
| RP11-11H9 |  | 2q21,21q11.2,12q11,14q11.2,15q11.2(UWMBT) | |
| RP11-122N11 |  | 8p23.1,4p16(UWMBT) | |
| RP11-125L9 |  | 21p13,22p13,13p13,14p13,15p13(UWMBT) | |
| RP11-125M18 |  | Yq11.2,8p23,6p25,4q26,16q24,5q35,19p13.3,3q29,6q27,9q34,10p11.1-10q11.1,20p13,1q41,11p15,1q43,1p36(UWMBT) | |
| RP11-125N16 |  | 7p14,7p11.2-7p12(UWMBT) | |
| RP11-126O12 |  | 1p12,1q21(UWMBT) | |
| RP11-12D5 |  | 6p24,Xp21(UWMBT) | |
| RP11-12E10 |  | 4q27,14q11.2,15q11.2,11p15.5(UWMBT) | |
| RP11-12p21 |  | 9p12-9p13,9q12-9q21,21p11.1-21p11.2,15p11.1-15p11.2,13p11.1-13q11,14p11.1-14q11.1,22p11.1-22p11.2(UWMBT) | |
| RP11-131O14 |  | 1p31.3,4q13.3-4q21(UWMBT) | |
| RP11-132H11 |  | Yp11.3,Xp22.3(UWMBT) | |
| RP11-134C5 |  | 2p11.1-2p11.2,21q11.1,22q11.1,2q21,14q11.1,15q11.1(UWMBT) | |
| RP11-139L10 |  | 6p11.1-6q11.1,11p15(UWMBT) | |
| RP11-139o21 |  | 15p11.1-15p11.2,2p11.1-2p11.2,1q12,21p11.1-21p11.2,13p11.1-13p11.2,7q36,14p11.1-14p11.2,22p11.1-22p11.2(UWMBT) | |
| RP11-13G8 |  | 10q21.1,13q14.1(UWMBT) | |
| RP11-140M6 |  | 2q21,22q11.1,14q11.1(UWMBT) | |
| RP11-141H6 |  | 11p11.1-11p11.2,11q14.3(UWMBT) | |
| RP11-145J12 |  | Xq21,Yp11.3(UWMBT) | |
| RP11-149i22 |  | 15p11.1-15p11.2,21p11.1-21p11.2,13p11.1-13p11.2,14p11.1-14p11.2,22p11.1-22p11.2(UWMBT) | |
| RP11-14K1 |  | 7p14-7p15,22q11.2,7p21,16p13.2(UWMBT) | |
| RP11-150n22 |  | 18p11.3,15p11.1-15p11.2,4q35,3q13.3-3q21,16p11.1-16p11.2,Yp11.1-Yp11.2,9p12-9p13,21p11.1-21p11.2,20q11.1-20q11.2,13p11.1-13p11.2,22p11.1-22p11.2,14p11.1-14p11.2,3p12-3p13(UWMBT) | |
| RP11-155J2 |  | 2p22,10q22,3p14,2q24(UWMBT) | |
| RP11-158H20 |  | 10p11.23,1p21.3-1p22.3(SC) | |
| RP11-15D18 |  | 11q22.1,11p15.5(UWMBT) | |
| RP11-15E1 |  | 9q12-9q21.11,14q12,2cen-2q11.2,9p11.1-9p12(SC) | |
| RP11-15K19 |  | 7q11.22,7p11.2(UWMBT) | |
| RP11-164D11 |  | 2p11.1-2p11.2,21q11.1,22q11.1,14q11.1,13q11,15q11.1,16p11.2(UWMBT) | |
| RP11-167C5 |  | 20p12-20p12.3,20cen,9p23-9p24(SC) | |
| RP11-171B15 |  | 1p31.1-1p31.3,1q44(SC) | |
| RP11-178M6 |  | Yq11.23,Yp11.2(UWMBT) | |
| RP11-179c7 |  | 9p11.1-9q12,2q21,21p11.1-21p11.2,22p11.1-22q11.1,15p11.1-15p11.2,14p11.1-14q11.1(UWMBT) | |
| RP11-17C13 |  | 7p14,8q21.3(UWMBT) | |
| RP11-17L12 |  | 9q12,1q12,21p11.2,13p11.2,3q11.2,3p12,22p11.2,21p13,14p11.2,13p13,22p13,15p11.2,14p13,4p11-4p12,15p13(UWMBT) | |
| RP11-182F22 |  | 16q24,19p13.3,1q41,1q43,1p36,7p22,Yq11.2,2q37,4q26,6p25,8p23,5q35,6q27,9q34,20p13,20q13.3(UWMBT) | |
| RP11-183L11 |  | 8q24.2,5q23(UWMBT) | |
| RP11-18D5 |  | 17q21,6p25,2q37,8p23,22q11.2,1q41,1p36(UWMBT) | |
| RP11-194F21 |  | 7q33-7q34,14q32.2(UWMBT) | |
| RP11-196H10 |  | 3q13.33,8q11.1(UWMBT) | |
| RP11-196I24 |  | 2q37.3,11p11.2-11p12(UWMBT) | |
| RP11-19M12 |  | 9p12,9q22.2,9q11-9q12(UWMBT) | |
| RP11-203D17 |  | 13q12.2-13q13.1,9p23(SC) | |
| RP11-216C10 |  | 7p13-7p14.3,20p11.23(SC) | |
| RP11-216O19 |  | 8p22,6p25,3p12,11q13,2q22,3q21,11p15,2p16,4p16(UWMBT) | |
| RP11-218E17 |  | 2q32.2-2q32.3,6q22(UWMBT) | |
| RP11-219D7 |  | 6q14,12q24.3(UWMBT) | |
| RP11-222F10 |  | 6q14,3q23-3q24(UWMBT) | |
| RP11-226I21 |  | 16q24,19p13.3,15q26,1q41,7p11.2,1q43,1p36,Yq11.2,2q37,4q26,6p25,8p23,5q35,3q29,6q27,9q34,10p11.1-10q11.1,20p13,22p11.1-22q11.1,20q13.3,11p15(UWMBT) | |
| RP11-226O1 |  | 7q22,7p14,9q12,12q14(UWMBT) | |
| RP11-227E4 |  | 20p11.2,8q12,13q14-13q21.1(UWMBT) | |
| RP11-228M15 |  | 15q11.2,15q12(UWMBT) | |
| RP11-231J1 |  | 3q12,1p33(UWMBT) | |
| RP11-232F14 |  | 7q11.22,5q23.1-5q23.2(UWMBT) | |
| RP11-235D20 |  | 2q21,22q11.1,14q11.1,9q12-9q13,15q11.1,9p11(UWMBT) | |
| RP11-236I4 |  | 7q35-7q36.1,11q22.3,14q21.2(UWMBT) | |
| RP11-238H10 |  | 8q22,2q24,6p23(UWMBT) | |
| RP11-23B24 |  | 9p12,Yq11.21,9q12,21p11.2,13p11.2,22p11.2,3q11.2,3p12,14p11.2,21p13,13p13,15p11.2,22p13,14p13,4p11-4p12,15p13(UWMBT) | |
| RP11-24F18 |  | Yp11.3,Yq11.22(UWMBT) | |
| RP11-251O17 |  | 9p11.2-9p13.1,9q21.11(SC);9p12,2q11.2,9q12-9q13(UWMBT) | |
| RP11-252A24 |  | 16q22-16q23,16p11.2,16p12(UWMBT) | |
| RP11-253I17 |  | 2q32,8p12(UWMBT) | |
| RP11-253O11 |  | 18q12.3,11p14.3(UWMBT) | |
| RP11-259I4 |  | Yq11.2,8p23,6p25,4q26,5q35,19p13.3,6q27,1q42,1q44(UWMBT) | |
| RP11-264E23 |  | 16p13.3,7q22,3q13.3-3q21,8p23,9q22,3p25-3p26,12p13,11q13,11p15,3p12-3p13,4p16,7p22(UWMBT) | |
| RP11-264I23 |  | 9p23,9q33(UWMBT) | |
| RP11-264M14 |  | 2p11.1-2q11.1,15q11.2,16p11.2(UWMBT) | |
| RP11-265F15 |  | 7q22,7q36.2(UWMBT) | |
| RP11-275F7 |  | Xq24,4p15.3-4p16(UWMBT) | |
| RP11-286G23 |  | 9p12,9q12(UWMBT) | |
| RP11-28A18 |  | 2q11.2,12q24.2(UWMBT) | |
| RP11-28P6 |  | Yp11.3,Xp22.3(UWMBT) | |
| RP11-291L22 |  | 16q24,Yq11.23,19p13.3,1q22,1q41,1q43,7p11.2,1p36.3,7p22,4q25,6p25,7q32,5q35,3q29,9q34.3,10p11.2,20p13,11p15.5(UWMBT) | |
| RP11-297J4 |  | 1q41,11q24(UWMBT) | |
| RP11-2A18 |  | 2q36,9p21.3-9p23(SC) | |
| RP11-2I9 |  | 9p21,11q22,1q32.2,12q24.2(UWMBT) | |
| RP11-2L10 |  | 12q12,3p25-3p26,1p36.2,13q33(UWMBT) | |
| RP11-301J16 |  | Yq12,13q11-13q12.13(SC) | |
| RP11-304L19 |  | 16p12,16p13.2(UWMBT) | |
| RP11-308K19 |  | 4q27-4q28.1,10p12.31-10p14(SC) | |
| RP11-30K9 |  | 9q31,20p,20q,2q21.2,22q11.2,16p,14q11.2,16q,13q31,15q21(UWMBT) | |
| RP11-311A12 |  | 3q26-3q26.32,9p23-9p24.3(SC) | |
| RP11-313I2 |  | 11p11.2,11q14(UWMBT) | |
| RP11-318K12 |  | 14q11.1-14q11.2,13q11-13q12.11,15q11.1-15q11.2,18cen,9p11.1-9p12,20q11.1-20q11.21,21q11.1-21q11.2,2cen,9q13-9q21.12,2q21.1-2q21.3,22q11.1-22q11.21(SC) | |
| RP11-324k20 |  | 16q24,Yq11.23,19p13.3,1q41,7p11.2,1q44,1p36.3,7p22,6p25,7q32,5q35,3q29,9q34.3,10p11.2,7q11.1-7q11.2,20p13,12p13,20q13.3,11p15.5(UWMBT) | |
| RP11-325K2 |  | 9p21.3-9p22.3,16p11.2-16p12.3(SC) | |
| RP11-327I12 |  | 2p24-2p25,10q25(UWMBT) | |
| RP11-32e24 |  | Yp11.2,Xq28(SC) | |
| RP11-33A10 |  | 2q14.3,11q24(UWMBT) | |
| RP11-33B1 |  | 16q24,19p13.3,3q29,7q11.1-7q11.2,9q34.3,10p11.2,1q41,4q26-4q27,1q43,7p11.2,1p36.3,11p15.5(UWMBT) | |
| RP1-133P16 |  | 7q21.3-7q22.1,6q26-6q27(SC) | |
| RP11-341D18 |  | 2p11.2-2p12,22cen,13cen(SC) | |
| RP11-343N15 |  | 1cen,1q32.2-1q41(SC) | |
| RP11-348G16 |  | 2p11.2,2q13(UWMBT) | |
| RP11-350E12 |  | 9q12,9p12-9p13.2,9q21.31-9q22.1(SC) | |
| RP11-350j20 |  | 6p21.2-6p21.33,6q24.1-6q25.1(SC) | |
| RP11-353N4 |  | 1p12,1q12-1q21.2,1p11.1,1p35.3-1p36.13(SC) | |
| RP11-354F21 |  | 22q11.2,14q11.2,2q21-2q22(UWMBT) | |
| RP11-358B14 |  | 16q22-16q23,16p11.2-16p12,16p12-16p13.1,18p11.1-18p11.2(UWMBT) | |
| RP11-361F15 |  | 6q24.1-6q24.3,6p21.1-6p21.31(SC) | |
| RP11-366C6 |  | Yq11.2,8p22-8p21,1q32(UWMBT) | |
| RP11-368B4 |  | 4p16.3,1q12(UWMBT) | |
| RP11-384I6 |  | 1q25.2-1q31.2,1q43-1q44(SC) | |
| RP11-384K6 |  | 16p13.3,16q24,19p13.3,1q41,1q43,7p11.2,7q11.2,1p36.3,7p22,Yq11.2,4q26,7q32,8p23,2q37.3,5q35,3q29,6q27,9q34.3,10p11.1,20p13,20q13.3,11p15(UWMBT) | |
| RP11-388B24 |  | 9p12-9p13.3,9q21.2-9q22.1(SC) | |
| RP11-38G7 |  | 12q24.33,12q13(UWMBT) | |
| RP11-38L15 |  | 10q23.1-10q23.33,10q11.21-10q11.23(SC) | |
| RP11-38M21 |  | 4q21,7q35(UWMBT) | |
| RP11-38P6 |  | 9p11.1-9p13.2,9q12-9q13(SC) | |
| RP11-390E9 |  | Xq21,Yp11.3(UWMBT) | |
| RP11-395E19 |  | 9p11.2-9p13.2,9q22.2,9q12-9q13(SC) | |
| RP11-395L14 |  | 16p13.3,19p13.3,15q26,17q25,19q13.4,2q14.1,1p36.3,7p22,9p12,6p25,8p23,9q13,3q29,9p24,6q27,9q34,20p13,12p13,20q13.3,11p15,22q13.3,10q26(UWMBT) | |
| RP11-3A5 |  | 11p11.1-11p11.2,11q14.3(UWMBT) | |
| RP11-400J9 |  | 9p12,10p11.2,2p11.1-2p11.2,10q11.2,1q12,22q11.2,9q12-9q13,14q11.2,7p11.2,15q11.2,16p11.2,7q11.2,17p11.2,17q11.2(UWMBT) | |
| RP11-402H23 |  | 1q42.11-1q42.3,7cen-7p11.2(SC) | |
| RP11-405L18 |  | 9p22.1-9p23,9p12-9p13.3(SC) | |
| RP11-407J2 |  | 7q22,7q11.2(UWMBT) | |
| RP11-413E6 |  | 16p13.3,15p11.1-15q11.1,3q13.3-3q21,Yp11.1-Yq11.1,3p25-3p26,1q21,9p12-9p13,9q12-9q13,4p16,7p22,7q22,8p23,4q35,19q13.3-19q13.4,9q22,20p11.1-20q11.1,10p12,21p11.1-21q11.1,21p13,12p13,11q13,13p13,22p11.1-22q11.1,11p15,14p13,13p11.1-13q11,15p13,14p11.1-14q11.(UWMBT) | |
| RP11-414G18 |  | 10p14,6q22.2-6q22.33(SC) | |
| RP11-421G7 |  | 2p14-2p15,13q21.1-13q21.2(SC) | |
| RP11-42E7 |  | Yq11.2,2q13-2q14,8p23,6p25,4q26,16q24,5q35,19q13,18q23,15q26,3q29,6q27,1q42,10p11.1-10q11.1,7p12,1p36(UWMBT) | |
| RP11-42O5 |  | 9q33,9q34.3(UWMBT) | |
| RP11-446K10 |  | 5q12,19q13.1-19q13.2(UWMBT) | |
| RP11-449B9 | RANDOM_SET | 22cen,13cen,14cen,15cen,16p11.2,10p11.1,2cen(UWMBT) | |
| RP11-449D1 | RANDOM_SET | Xq12,4q21,4q31.3-4q32(UWMBT) | |
| RP11-449D23 | RANDOM_SET | 16p12-16p13.1,16p11.2(UWMBT) | |
| RP11-449K24 |  | 16p11.2,16p12(UWMBT) | |
| RP11-44F14 |  | 16q12.1,5q13(LANL) | |
| RP11-44K8 |  | 10q21.3,9p23(UWMBT) | |
| RP11-451G6 | RANDOM_SET | 2p11.1-2q11.1,22cen,1q12,16cen,14p11.1-14q11.1,21cen(UWMBT) | |
| RP11-451O12 | RANDOM_SET | 2q31,7p12(UWMBT) | |
| RP11-451O2 | RANDOM_SET | 1p13,6p21.3(UWMBT) | |
| RP11-452L16 | RANDOM_SET | 2p11.1-2p11.2,2q11.1-2q11.2,15q11.1-15q11.2,16p11.1(UWMBT) | |
| RP11-453N3 |  | 9p12,9q12,10p11.1-10p11.2,2p11.1-2q11.1,14p11.1,22q11.1,14p11.2,16p11.1,15q11.1(UWMBT) | |
| RP11-456N16 |  | 16q23,19p13.1,16p12(UWMBT) | |
| RP11-462G22 |  | 4q35,14p11.2(UWMBT) | |
| RP11-463M3 |  | 13q12.11-13q12.2,6p24.1-6p25.3(SC) | |
| RP11-464D20 |  | 17q23,7q31.3-7q32(UWMBT) | |
| RP11-466N15 |  | 16q24,19q13,19p13.3,1q42,7q11.1,1p36,1q44,7p12,Yq11.2,4q26,2q37,6p25,8p23,5q35,3q29,6q27,9q34,10p11.1-10q11.1,11p15,20q13.3(UWMBT) | |
| RP11-467H10 | RANDOM_SET | 7q22,7q11.23(UWMBT) | |
| RP11-46M12 |  | 15q15,15q25(UWMBT) | |
| RP11-473N15 |  | Xq27.1-Xq27.3,Xq22.3-Xq24(SC) | |
| RP11-479C13 |  | 7q22,7q11.2,7p22(UWMBT) | |
| RP11-47F2 |  | Yq11.2,7q31,5p15.3,8p23,6p25,2q37,16q24,9q31,19p13.3,3q29,1q41,11p15,1q43,1p36(UWMBT) | |
| RP11-480c16 |  | 9p13,8q21.3,2q11.2,9q13,9q22,2q14.3(UWMBT) | |
| RP11-497C14 |  | Yq11.2,15q25(UWMBT) | |
| RP11-497H16 |  | 6p25,5q12,5p14(UWMBT) | |
| RP1-149A16 |  | 22q13-22q13.2,22q12(SC) | |
| RP11-4E17 |  | 9q21.1,5q35(UWMBT) | |
| RP11-509A17 |  | 17q25.3,2p11.2,1q21,17q22-17q23,2p24,9p12,9q12,9q13,10p11.1-10p12,2p11.1-2p11.2,22q11.2,13q12,2q11.2-2q12,14q11.2,15q11.2,16p11.2(UWMBT) | |
| RP11-50F16 |  | 17q12,17q23(UWMBT) | |
| RP11-528K16 |  | 16q24,16p11.2(LANL) | |
| RP11-542J23 |  | 1p12,1q12,19p13.3,1p36.1,3p25-3p26(UWMBT) | |
| RP11-546N8 |  | 11p11.2,11q14(UWMBT) | |
| RP11-553E2 |  | 9p13-9p21.1,9q22-9q32(SC) | |
| RP11-554P16 |  | Xq26.3-Xq27.1,Xp11.1-Xp11.3(SC) | |
| RP11-555k2 |  | 9p12,4q35,14p11.1-14p11.2,9q12,4q28,21q11.1-21q11.2,13p11.1-13p11.2(UWMBT) | |
| RP11-55C21 |  | 10q11.2,10q22.2(UWMBT) | |
| RP11-577O18 |  | 16p13.3,7q22,3q13.3-3q21,2q24-2q31,8p23,9q22,3p25-3p26,10p15,13q21,11p15,2p12-2p13,7q11.2,11q12-11q13,3p12-3p13,4p16,7p22(UWMBT) | |
| RP11-57D15 |  | 7q21.3,4q28(UWMBT) | |
| RP11-57F22 |  | 6q16.1,12q21(UWMBT) | |
| RP11-57L15 |  | 14q24,12q21(UWMBT) | |
| RP11-58L1 |  | 3q13.3-3q21,8p23,9q22,3p25-3p26,10p15,11q13.4,3p12-3p13,4p16,11p15.5(UWMBT) | |
| RP11-597A11 |  | 14q11.1-14q11.2,15q11.1-15q11.2,2q21,22q11.2(UWMBT) | |
| RP11-597O11 |  | 16p13.3,7q22,3q13.3-3q21,2q24-2q31,8p23,9q22,3p25-3p26,10p15,13q21,11p15,2p12-2p13,11q12-11q13,3p12-3p13,4p16,7p22(UWMBT) | |
| RP11-598C10 |  | 1q22-1q24.3,10q21.1-10q21.2(SC) | |
| RP11-599L24 |  | 16p13.3,7q22,3q13.3-3q21,2q24-2q31,8p23,9q22,3p25-3p26,2p13,10p15,13q21,11p15,11q12-11q13,3p12-3p13,4p16,7p22(UWMBT) | |
| RP11-59J12 |  | 8p11.1-8p11.2,1q43(UWMBT) | |
| RP11-5M23 |  | 15q14-15q15,6q12-6q13(UWMBT) | |
| RP11-602C12 |  | 16p13.3,3q13.3-3q21,2q24-2q31,3p25-3p26,7p22,4p16,6q26-6q27,5p15.3,7q22,8p23,9q22,21q22.1,10p15,13q21,2p12-2p13,11p15,3p12-3p13,11q12-11q13(UWMBT) | |
| RP11-604J16 |  | 16p13.3,7q22,3q13.3-3q21,2q24-2q31,8p23,9q22,3p25-3p26,10p15,13q21,11p15,14q21,11q12-11q13,3p12-3p13,4p16,7p22(UWMBT) | |
| RP11-604O2 |  | 13q21,13q13(UWMBT) | |
| RP11-606K14 |  | 16p13.3,7q22,3q13.3-3q21,2q24-2q31,8p23,9q22,3p25-3p26,10p15,13q21,11p15,2p12-2p13,11q12-11q13,3p12-3p13,4p16,7p22(UWMBT) | |
| RP11-608N20 |  | 16p13.3,7q22,3q13.3-3q21,2q24-2q31,8p23,9q22,3p25-3p26,12p13,11q13,10p15,13q21,2p12-2p13,14q21,13q13,3p12-3p13,4p16,7p22(UWMBT) | |
| RP11-611N5 |  | 16p13.3,3q13.3-3q21,2q24-2q31,3p25-3p26,3q21,4p16,7p22,7q22,8p23,9q22,10p15,11q13,12p13,2p12-2p13,13q21,14q21,13q13,3p12-3p13(UWMBT) | |
| RP11-61B21 |  | 2p23-2p24,20q13.1(UWMBT) | |
| RP11-61D13 |  | 16p11.2-16p12,16p13.1-16p13.2(UWMBT) | |
| RP11-61I9 |  | 4q21,3p26(UWMBT) | |
| RP11-620F19 |  | 16q24,19p13.3,15q26,7p11.1,1q42,7q11.1,1p36,1q44,7p22,4q26,6p25,7q32,8p23,5q35,3q29,6q27,9q34,10p11.1-10q11.1,20p13(UWMBT) | |
| RP11-622M13 |  | 11p11.2,11q12,11q14(UWMBT) | |
| RP11-626D6 |  | 16p13.3,7q22,3q13.3-3q21,2q24-2q31,8p23,9q22,3p25-3p26,10p15,21q22,13q21,11p15,2p12-2p13,11q12-11q13,3p12-3p13,4p16,7p22(UWMBT) | |
| RP11-630O8 |  | 3q21,21q22,13q21,14q21,13q13(UWMBT) | |
| RP11-63M17 |  | 12p11.2-12p12.1,12q12-12q13.1(UWMBT) | |
| RP11-642E22 |  | 4q26,19p13.2,16q24,3q29,9q34,10p11.2,20p13,1q41,1q43,7p11.2,1p36,11p15.5(UWMBT) | |
| RP11-664F7 |  | Yq11.2,5q33,4q26,5q35,19p13.3,9p24,3q29,7p11.1,1q42,1p36,6p22,6q12,7p22(UWMBT) | |
| RP11-669E14 |  | 4q26,16q24,5q35,3q29,1q41,9q34,1q43,7q11.2,7p12,1p36,11p15.5,10p11.2,7p22(UWMBT) | |
| RP11-66G15 |  | 15q26.2-15q26.3,13q31.1-13q31.3(SC) | |
| RP11-66N16 |  | 12q22-12q23.1,1q24(UWMBT) | |
| RP11-671B24 |  | 16q24,19q13,19p13.3,1q41,1q42,7p12,1p36,7p22,Yq11.2,2q37,4q26,6p25,8p23,5q35,3q29,9q34,10p11.1-10q11.1,20p13,20q13.3,11p15(UWMBT) | |
| RP11-67O5 |  | 7p11.2,7q11.2(UWMBT) | |
| RP11-688n14 |  | 19p13.2,16q24,15q26,1q41,7p11.2,1q43,7q11.2,7p12,1p36,4q32,7p22,Yq11.2,4q26,6p25,7q32,8p23,2q37.3,5q35,3q29,6q27,9q22,9q34.3,10p11.2,20p13,20q13.3,11p15.5(UWMBT) | |
| RP11-691I13 |  | 9p13.1-9p21.1,10p11.21-10p12.1(SC) | |
| RP11-69E18 |  | 9p13,9q13,9q22.3(UWMBT) | |
| RP1-170A21 |  | 22q12.3,22q13.3(SC) | |
| RP11-730J6 |  | 19p13.2,15q26.3,1q41,7p11.2,1q43,7q11.2,1p36,7p22,4q34.1,Yq11.2,6p25,8p23,2q37.3,5q35,7q33,3q29,4q28,9p24,6q27,9q34.3,10p11.2,20p13,20q13.3,11p15.5(UWMBT) | |
| RP11-756A22 |  | 13q12.11-13q12.3,13p11.1-13p11.2(SC) | |
| RP11-758A3 |  | Xq25,Xp11.3-Xp21.1(SC) | |
| RP11-77p19 |  | 2p11.1-2p11.2,21q11.1,22q11.1,13q11,14q11.1,21q21,2q22,15q11.1,18p11.2(UWMBT) | |
| RP11-78P11 |  | 7q22,7q11.23(UWMBT) | |
| RP11-793L10 |  | 6q16.2-6q21,5q34-5q35.2,21q22.3(SC) | |
| RP11-799H15 |  | 8p23,5q35,19p13.3,15q26,3q29,6q27,9q34.3,1p36,11p15.5(UWMBT) | |
| RP11-79A23 | RANDOM_SET | 15cen,16cen,7cen,17cen,18cen,9cen,1cen,10cen,2cen,21cen,22cen,13cen,14cen(UWMBT) | |
| RP11-79A8 | RANDOM_SET | 1p12,1q21,1p36.2(UWMBT) | |
| RP11-79a9 | RANDOM_SET | 9q11,2q11.1-2q11.2,9q12-9q13,14q12,9p11(UWMBT) | |
| RP11-79c9 | RANDOM_SET | 16p12,16p11.2(UWMBT) | |
| RP11-79E14 | RANDOM_SET | 1q44,Yp11.2(UWMBT) | |
| RP11-79G14 | RANDOM_SET | 11p11.2,11q12(UWMBT) | |
| RP11-79G8 | RANDOM_SET | 5q21,4p15.3-4p16(UWMBT) | |
| RP11-79h1 | RANDOM_SET | 9q12,4q26,9p11.2(UWMBT) | |
| RP11-79H21 | RANDOM_SET | Yq11.2,Yp11.2(UWMBT) | |
| RP11-79H3 | RANDOM_SET | 2q21,21q22(UWMBT) | |
| RP11-79I20 | RANDOM_SET | 2p24,6p22(UWMBT) | |
| RP11-7E21 |  | 7q32.1,7p15.2(UWMBT) | |
| RP11-802L12 |  | 22q11.1-22q11.23,6p22.3-6p23(SC) | |
| RP11-816C16 |  | 8p23,5q35,19p13.3,15q26,3q29,6q27,9q34.3,1p36,11p15.5(UWMBT) | |
| RP11-81J13 | RANDOM_SET | 16q22-16q23,16p11.2,16p12,18p11.2(UWMBT) | |
| RP11-81J17 | RANDOM_SET | 18q12.1,2q21(UWMBT) | |
| RP11-81J23 | RANDOM_SET | 7q22,7q11.23(UWMBT) | |
| RP11-841E22 |  | 8p22-8p23,12q13(UWMBT) | |
| RP11-84N24 |  | 2p11.1-2q11.1,9p12-9p13,9q12-9q13,14q12(UWMBT) | |
| RP11-85c8 |  | 15p11.1-15p11.2,Yp11.1-Yp11.2,1q12,2p13,3q11.2,4p16.3,9p12,9q12,21p11.1-21p11.2,2q11.1-2q11.2,20q11.1-20q11.2,21p13,13p13,13p11.1-13p11.2,22p11.1-22p11.2,14p13,15p13,4p11-4q11,14p11.1-14p11.2(UWMBT) | |
| RP11-86E13 |  | 17q24,4q21,12q14(UWMBT) | |
| RP11-86F24 |  | 1p21,13q12-13q13.3(SC) | |
| RP11-88B4 | RANDOM_SET | 7p11.2,7q11.21,7p22(UWMBT) | |
| RP11-88D4 | RANDOM_SET | 11q14.1,13q13(UWMBT) | |
| RP11-88J14 | RANDOM_SET | 21p13,22p13,13p13,14p13,15p13(UWMBT) | |
| RP11-88L8 | RANDOM_SET | 9q34.1,18p11.1-18q11.1(UWMBT) | |
| RP11-88P2 | RANDOM_SET | 3q13.3-3q21,3p12,3p25(UWMBT) | |
| RP11-89B7 | RANDOM_SET | 16p11.2,16p12(UWMBT) | |
| RP11-89C20 | RANDOM_SET | 15cen,1q12,3p12,3q11.2,4cen,9p12,9q12,Ycen,21p13,13p13,20cen,22p13,14p13,21cen,13cen,15p13,22cen,14cen(UWMBT) | |
| RP11-89C8 | RANDOM_SET | 15cen,9p12,9q12,1q12,20cen,21cen,16p11.2,22cen,13cen,14cen(UWMBT) | |
| RP11-89D14 | RANDOM_SET | 9p12,9q12,18p11.1-18p11.2,21p11.2,1q12,2q11.2,22p11.2,2q21,13p11.2,21p13,14p11.2,22p13,20cen,15p11.2,13p13,14p13,3cen,15p13,4p11-4p12(UWMBT) | |
| RP11-89d23 | RANDOM_SET | 16p13.3,9p12,9q12,6p25,19p13.3,15q26,9p24,3q29,20p13,20q13.3,12p13.3,2q13-2q14.1,10q26,11p15.5,1p36.3(UWMBT) | |
| RP11-89d24 | RANDOM_SET | 5q15-5q21,6p11.1-6p11.2,5p13,5q12,5p14(UWMBT) | |
| RP11-89F3 | RANDOM_SET | 1p12,1q12,1p36.2(UWMBT) | |
| RP11-89H9 | RANDOM_SET | 15q14,15q24,15q11.2-15q12,10p11.2(UWMBT) | |
| RP11-89I18 | RANDOM_SET | 9p12,4q28(UWMBT) | |
| RP11-89J1 | RANDOM_SET | 7p11.2,7q11.21,7p22(UWMBT) | |
| RP11-89L14 | RANDOM_SET | 9p12,9q12,2p11.2,1q12,21q11.2,2q12,22q11.2,14p11.2,10cen,15p11.2,14q11.2,16p11.2,15q11.2,12cen,13cen,4p16(UWMBT) | |
| RP11-89l19 | RANDOM_SET | 9p12,14p11.1-14p11.2,9q12,15p11.1-15p11.2,1p12,3p11-3q11.1,21p13,21p11.1-21p11.2,22p13,13p13,14p13,22p11.1-22p11.2,4p11-4p12,13p11.1-13p11.2,15p13(UWMBT) | |
| RP11-89L8 | RANDOM_SET | 15q26.2,13q31(UWMBT) | |
| RP11-89M6 | RANDOM_SET | Yp11.3,Xq21.2(UWMBT) | |
| RP11-89n13 | RANDOM_SET | 22p12-22p13,13p12-13p13,14p12-14p13,21p12-21p13,15p12-15p13(UWMBT) | |
| RP11-8I12 |  | 20q11.2,12p13,22q13.3(UWMBT) | |
| RP11-90D23 | RANDOM_SET | 2p11.2,2q12,6q26(UWMBT) | |
| RP11-90f19 | RANDOM_SET | 22q11.1-22q11.2,9p12,13q11-13q12,9q12,14q11.1-14q11.2,10p11.1-10p11.2,15q11.1-15q11.2,16p11.1-16p11.2,2p11.1-2q11.1,1q12,2p24(UWMBT) | |
| RP11-90G15 | RANDOM_SET | 9q21.1,9p11.1-9q12,19q13.1,4q28,21cen,13cen,14cen(UWMBT) | |
| RP11-90H13 | RANDOM_SET | 1q12,2cen,7p11.2,7q11.2,9p11.2,9q11,Yq11.2,10p11.2,10q11.2,13p11.2,21q11.2,14p11.2,22q11.2,15p11.2,15q11.2,16p11.2,16q11.2(UWMBT) | |
| RP11-90K10 |  | 2q36-2q37,7q11.2-7q21(UWMBT) | |
| RP11-90P13 | RANDOM_SET | 5q22-5q23,7p22(UWMBT) | |
| RP11-90p3 | RANDOM_SET | 9q11,19p12,19q12,10p11.2,2p11.2,1q12,2q11.2,22q11.2,14p11.2,15p11.2,16p11.2,15q11.2,9p11(UWMBT) | |
| RP11-91a10 | RANDOM_SET | 21p11.1-21q11.1,13p11.1-13q11,14p11.1-14q11.1(UWMBT) | |
| RP11-91A12 | RANDOM_SET | 19q13.2,11q13,2p16,21cen,13cen,14cen(UWMBT) | |
| RP11-91a8 | RANDOM_SET | 21p11.1-21q11.1,13p11.1-13q11(UWMBT) | |
| RP11-91B9 | RANDOM_SET | 20q12,1q32(UWMBT) | |
| RP11-91D19 | RANDOM_SET | Yp11.3,Xp22.3(UWMBT) | |
| RP11-91D5 | RANDOM_SET | Yp11.3,Xp22.3(UWMBT) | |
| RP11-91e5 | RANDOM_SET | 15p11.1-15p11.2,15q15(UWMBT) | |
| RP11-91F5 | RANDOM_SET | 8p23,12p13,11q13,11p15,4p16(UWMBT) | |
| RP11-91G11 | RANDOM_SET | 1p12,1q12,5q11.2,1p36.1(UWMBT) | |
| RP11-91G24 | RANDOM_SET | Xq21,15cen,7q36,21p11.1-21q11.1,12q21,22p11.1-22q11.1,2cen,13p11.1-13q11,14cen(UWMBT) | |
| RP11-91i10 | RANDOM_SET | 2p11.1-2q11.1,15cen,Yq12,13p11.1-13q11,21cen,22cen,14cen(UWMBT) | |
| RP11-91i14 | RANDOM_SET | 15p11.1-15q11.1,13p11.1-13q11,21cen,14p11.1-14q11.1,22cen(UWMBT) | |
| RP11-91I4 | RANDOM_SET | 1p13.2,1q12-1q21,1p35(UWMBT) | |
| RP11-91K21 | RANDOM_SET | 3p11-3q11.1,6p12,6q12(UWMBT) | |
| RP11-91k22 | RANDOM_SET | 15cen,9p13,9q13,13p11.1-13q11,21cen,22cen,2q21-2q22,14cen,18p11.2(UWMBT) | |
| RP11-91K3 | RANDOM_SET | 9q13,10p11.2,10q11.2,2p11.1-2p11.2,1q12,15q11.2,16p11.2,7p11.2,7q11.2,4p16(UWMBT) | |
| RP11-91L21 | RANDOM_SET | 16p12,1q31.3-1q32.1(UWMBT) | |
| RP11-91M20 | RANDOM_SET | 16p13.3,5p15.3,2q22-2q23,8p23,3q13.3-3q21,3p25-3p26,12p13,2p12-2p13,11p15,3p12-3p13,10q26,11q12-11q13,4p16,7p22(UWMBT) | |
| RP11-91N17 | RANDOM_SET | 10q21.1,21q22(UWMBT) | |
| RP11-94M13 |  | 8p21.3,8q24.2(UWMBT) | |
| RP11-94N6 |  | 14q24.2,7q21.3(UWMBT) | |
| RP11-95A21 |  | 1p21,5q14(UWMBT) | |
| RP11-95F11 |  | 6p25,15q25,5q35,3q29,15q26.3,6q27,19q13.4,15q12,11p15.5,1p36.3(UWMBT) | |
| RP11-95J1 |  | 4q28,4q12(UWMBT) | |
| RP11-96D2 |  | 16p13.3,8p23.2,3p12,12p13.2,3q21,11q13,11p15.4,4p16.2(UWMBT) | |
| RP11-96F3 |  | 6q25,20q13.3,12p13.3(UWMBT) | |
| RP1-196P3 |  | 20p13,6q16.3-6q21(SC) | |
| RP11-97H6 |  | 11p11.11-11p11.12,7p12(UWMBT) | |
| RP11-98M19 |  | 4q25-4q26,6p23(UWMBT) | |
| RP11-9B4 |  | 7q32.1,7p14-7p15.1(UWMBT) | |
| RP11-9G4 |  | 17q22,17p13.1(UWMBT) | |
| RP1-210G8 |  | 6q11.2-6q12,6q25.2-6q26(SC) | |
| RP13-118G24 |  | Xp11.23-Xp11.4,1q32.1-1q32.2(SC) | |
| RP13-136L2 |  | Yp11.31-Yp11.32,Xq21.31-Xq21.33(SC) | |
| RP13-138P15 |  | Yp11.31-Yp11.32,Xq21.2-Xq21.33(SC) | |
| RP13-13L21 |  | 13q11-13q12.2,Xq26.1-Xq27.2(SC) | |
| RP13-140E4 |  | Yp11.31-Yp11.32,Xq21.2-Xq21.33(SC) | |
| RP13-159P11 |  | Yp11.31-Yp11.32,Xq21.2-Xq21.33(SC) | |
| RP13-17E1 |  | Yp11.31-Yp11.32,Xq21.31-Xq21.33(SC) | |
| RP13-212L9 |  | Yp11.31-Yp11.32,Xq21.31-Xq21.33(SC) | |
| RP13-21D1 |  | Yp11.31-Yp11.32,Xq21.2-Xq21.33(SC) | |
| RP13-258O15 |  | Yp11.31-Yp11.32,Xq21.2-Xq21.33(SC) | |
| RP13-310B24 |  | Yp11.31-Yp11.32,Xq21.31-Xq21.33(SC) | |
| RP13-348B13 |  | Yp11.31-Yp11.32,Xq21.2-Xq21.33(SC) | |
| RP13-362E11 |  | Yp11.31-Yp11.32,Xq21.2-Xq21.33(SC) | |
| RP13-448I11 |  | Yp11.31-Yp11.32,Xq21.31-Xq21.33(SC) | |
| RP1-34P24 |  | 22q13.1,22q13.3,22q13.2-22q13.33(SC) | |
| RP13-73G13 |  | Yp11.31-Yp11.32,Xq21.2-Xq21.33(SC) | |
| RP13-88F20 |  | Yp11.2-Yp11.32,Xq21.2-Xq21.33(SC) | |
| RP13-94p8 |  | Yp11.31-Yp11.32,Xq21.31-Xq21.33(SC) | |
| RP1-57A13 |  | Xq24,2q33(SC) | |
| RP3-322E17 |  | 1p36.11-1p36.23,1p12-1p13.2,1q12-1q21.2(SC) | |
| RP3-368B9 |  | 9q12,9p11.1-9p12,4p16.1-4p16.3(SC) | |
| RP3-401D24 |  | 3cen-3p11.2,6cen-6q12(SC) | |
| RP3-404P13 |  | 12q12-12q13.13,22q13.2(SC) | |
| RP3-414L4 |  | 10q23,6q25.1-6q26(SC) | |
| RP3-437C15 |  | 16cen,6cen-6p12.1(SC) | |
| RP3-447N6 |  | Xq24-Xq25,Xq22,Xp11,Xq21.1(SC) | |
| RP3-462D8 |  | 11q13.1-11q13.4,22q11.21-22q12.1(SC) | |
| RP3-481A17 |  | Xq24-Xq25,Xq21-Xq22,Xp11,Xq21.1(SC) | |
| RP3-496C20 |  | 22q12.3-22q13.1,22q13.1(SC) | |
| RP4-538N4 |  | 1p21.1-1p21.3,1p13.1-1p13.3(SC) | |
| RP4-555J6 |  | 1p12-1p13.2,1q12-1q21.3(SC) | |
| RP4-565E6 |  | 1p12-1p13.2,1p22.2-1p31.1,1q12-1q21.1,1p35.1-1p36.23(SC) | |
| RP4-579N16 |  | 22p11.1-22cen,22cen-22q11.21,22q13.2-22q13.33(SC) | |
| RP4-609N19 |  | 2q13-2q14,16q24,15q26.1,19p13.3,1q41,7p11.2,1q43,1p36,Yq11.2,6p25,2q37,4q26,8p23,5q35,3q29,9p24,6q27,9q34,10p11.1-10q11.1,20p13,22p11.1-22q11.1,20q13.3,11p15(UWMBT) | |
| RP4-610C12 |  | 20p11.1-20p11.21,9q11-9q12,20q11.1-20q11.22,9p11.1-9p12(SC) | |
| RP4-636L22 |  | 1p12-1p13.2,1q12-1q21.2,1q32.1-1q32.3(SC) | |
| RP4-640F2 |  | 7q11.22-7q21.11,1p22.2-1p31.1,1p32.1-1p32.3,6p22.3-6p24.1(SC) | |
| RP4-646P11 |  | 1p12-1p31.2,1q12-1q21.2,1p36.11-1p36.31(SC) | |
| RP4-651A20 |  | 7cen,20q13.13-20q13.32(SC) | |
| RP4-679C16 |  | 1p36.13-1p36.31,1q22-1q23.3(SC) | |
| RP4-686J16 |  | 10q25.3-10q26.2,10q23.31-10q24.33,1p32.3-1p33(SC) | |
| RP4-691G10 |  | 1q12-1q21.2,1cen-1p13.1,1p36.11-1p36.13(SC) | |
| RP4-710H13 |  | 20q11.2,20q13.11-20q13.2(SC) | |
| RP4-725K1 |  | 1q12-1q21.2,1cen(SC) | |
| RP4-746H2 |  | 20q12,20p11.1-20p11.23(SC) | |
| RP4-760C5 |  | 7p11.1-7p11.2,20p11.1-20p11.22,20q11.1-20q11.22(SC) | |
| RP4-791M13 |  | 1p36.13-1p36.31,1cen,1q22-1q23.2(SC) | |
| RP4-796I17 |  | 4q35.1-4q35.2,22q13.2-22q13.32(SC) | |
| RP4-798A10 |  | 1p36.11-1p36.23,1q21.1-1q21.3(SC) | |
| RP4-800D18 |  | 1p21.1-1p21.3,1p31.1-1p31.3(SC) | |
| RP5-1074I8 |  | Xq13.1-Xq21.1,19q13.13-19q13.31(SC) | |
| RP5-1077B9 |  | 1q21.1,1p36.11-1p36.32(SC) | |
| RP5-1093P10 |  | 1q12-1q21.2,1p36.2,1p11.1-1p12(SC) | |
| RP5-1099D15 |  | 20p12.1-20p12.3,6q22.33-6q24.1(SC) | |
| RP5-1129d5 |  | Yq11.2,15q26.1(UWMBT) | |
| RP5-1182A14 |  | 1p36.13-1p36.31,1q21.1-1q21.3(SC) | |
| RP5-831C21 |  | 20p11.1-20p11.21,22q11.21(SC) | |
| RP5-850N15 |  | 20p11.21-20p11.23,10p11.22-10p12.33(SC) | |
| RP5-855D21 |  | 16q24,19p13.3,15q26,1q42,1p36,1q44,8p23,2q37,4q26,6p25,5q35,3q29,6q27,9q34,20p13,11p15(UWMBT) | |
| RP5-891l14 |  | 1q41,7p11.1,1q43(UWMBT) | |
| RP5-961K14 |  | Xq22.1-Xq23,1p31.3-1p32.3(SC) | |
| RP5-998H6 |  | 12q13.11-12q13.12,20q13.1(SC) | |
| RP5-998N21 |  | 1q12-1q21.1,1cen,1q32.1-1q32.3(SC) | |
